# Towards values‐based healthcare for inherited metabolic disorders: An overview of current practices for persons with liver glycogen storage disease and fatty acid oxidation disorders

**DOI:** 10.1002/jimd.12555

**Published:** 2022-09-30

**Authors:** Annieke Venema, Fabian Peeks, Alessandro Rossi, Emmalie A. Jager, Terry G. J. Derks

**Affiliations:** ^1^ Department of Metabolic Diseases, Beatrix Children's Hospital, University Medical Centre Groningen University of Groningen Groningen The Netherlands; ^2^ Department of Translational Medicine, Section of Pediatrics University of Naples “Federico II” Naples Italy

**Keywords:** fatty acid oxidation, glycogen storage disease, inborn errors of metabolism, value‐based healthcare

## Abstract

Value‐based healthcare (VBHC) intends to achieve better outcomes for patients, to improve quality of patient care, with reduced costs. Four dimensions define a model of intimately related value‐pillars: personal value, allocative value, technical value, and societal value. VBHC is mostly applied in common diseases, and there are fundamental challenges in applying VBHC strategies to low volume, high complex healthcare situations, such as rare diseases, including inherited metabolic disorders. This article summarizes current practices at various academical domains (i.e., research, healthcare, education, and training) that (aim to) increase values at various value‐pillars for persons with liver glycogen storage diseases or fatty acid oxidation disorders and their families. Future perspectives may include facilitating virtual networks to function as integrated practice units, improving measurement of outcomes, and creating information technology platforms to overcome the ethical, legal, societal, and technical challenges of data sharing for healthcare and research purposes.

## 1 INTRODUCTION

Many sectors of modern society are under great financial pressure, and research, healthcare, and education are no exceptions. By organizing healthcare according to the needs and preferences of individual patients, value‐based healthcare (VBHC) intends to achieve better outcomes for patients and hence, improving quality of patient care at reduced costs.^1^ Traditionally, the four principles of VBHC include (a) patient view, (b) medical view, (c) cost view, and (d) organization view. More recently this traditional definition of VBHC has been modulated by the Expert Panel on effective ways of investing in healthcare, by envisioning a more comprehensive concept of VBHC built on four value‐pillars: appropriate care to achieve patients' personal goals (personal value), achievement of best possible outcomes with available resources (technical value), equitable resource distribution across all patient groups (allocative value), and contribution of healthcare to social participation and connectedness (societal value).^2^

VBHC is mostly applied to high volume, low complexity health conditions, such as primary care or diabetes mellitus. To the best of our knowledge, there is no medical‐scientific literature on the VBHC approach in rare inherited metabolic disorders (IMDs), but healthcare models have been described for rare disorders (RDs). In accordance with the VBHC principles, Augustine et al. stated that traditional healthcare that involves local delivery of care, does not easily meet the needs of families confronted with RDs.^3^ RDs often require multidisciplinary expert care, and individually affect small numbers of patients all over the world. These authors propose a new healthcare model for patients with RDs, focusing on telemedicine, integrating care and research, and improving collaborations between patients, clinicians, and researchers. Integrating care and research, monitoring from home and standard outcome measures are important to manage RDs. Fantini and Vaccaro performed a literature review on VBHC related to RDs and stated that health should not only be considered the absence of illness, but a search for well‐being in the diverse conditions of life and according to various individual constitutions.^4^ This is also applicable to IMDs, in which the disorder is always present, not meaning that the individual's goals in life cannot be accomplished. IMDs severely affect not only the lives of the patient, but also their families and carers and have a substantial impact at various domains, such as political, economic, socio‐cultural, technological, legal, and ethical.^5^ Therefore, the traditional application of VBHC principles to IMDs seems limited if it is based on a costs/benefits ratio only. Table 1 presents difficulties in applying VBHC strategies to IMDs, in addition to the difficulties as mentioned by Fantini and Vaccaro.

**TABLE 1:** Difficulties in applying VBHC strategies to IMDs

Difficulties for VBHC in rare diseases	
1	The scope and capacity of most registries and databases are limited to the pure medical and clinical discourses.^a^
2	The knowledge of most RDs is insufficient (“orphan diseases”).^a^
3	The longitudinal data collections are scarce.^a^
4	The outcomes of treatment and care are diverse and difficult to quantify.^a^
5	The diagnosis is difficult and often delayed.^a^
6	The development of therapeutics and treatments is often fragmented and slow.^a^
7	The specialized and coordinated medical care is scarse and expensive, because of its complexity and multidimensionality.^a^
8	The standards of care for treatment and rehabilitation are not evidence based because health research is necessarily done at small scale.^a^
9	Healthcare for RD patients is traditionally organized in university hospitals, accommodating fundamental and clinical research, expert healthcare services, education and teaching, and valorization. The RD patients usually do not live close to expert HCPs, but are scattered across populations and consequently most patients are confronted with HCPs who lack expertise on their disease.
10	There is large heterogeneity in clinical, dietary and biomedical features among RD patients with identical subtypes and sometimes even genotypes.
11	Traditional biomedical outcome parameters need to be carefully balanced against psychosocial well‐being and quality of life, and hence patient reported outcome measures (PROMs) and patient reported experience measures (PREMs).
12	Monitoring and management guidelines and care pathways differ in different countries and sometimes even within the same country, causing unwanted variations in diagnosis, treatment, and outcomes.
13	Few registered treatments or orphan drugs are available.
14	There is inadequate exchange of clinical and scientific expertise. Currently, knowledge exchange between HCPs often takes place on a single patient basis, rather than being structural and amplifying.
15	There are no standard operating procedures in place for expert second opinions, to transfer RD expertise to affected families and local HCPs.

Abbreviations: IMDs, inherited metabolic disorders; RDs, rare diseases; VBHC, value‐based healthcare.

^a^ As mentioned in Fantini and Vaccaro.

Given the need to adhere to medically prescribed, strict diets, the high level of self‐management, and the use of medical devices at home for self‐monitoring, both liver glycogen storage diseases (GSDs) and fatty acid oxidation disorders (FAODs) can serve as exemplary IMDs to adapt, adopt, and improve VBHC elements. GSDs are characterized by an abnormal synthesis or degradation of glycogen, because of a defective enzyme or transporter. Liver GSD is a collective name for GSD subtypes 0, Ia, Ib, III, IV, VI, IX, and XI.^6^ The FAODs form another group of IMDs of energy metabolism, causing a variety of symptoms and signs in organs that depend on energy production by the carnitine shuttle and subsequent mitochondrial fatty acid oxidation, such as the heart, skeletal muscle, liver, and brain.^7^

In this review, we first showcase some recent practices for persons with GSD or FAOD that have aimed to increase value at one or more value‐pillars. These practices have taken place at different academic domains (i.e., research, healthcare, education, and training). Table 2 presents the practices mentioned in this article and which of the value‐pillars are most directly impacted. The open cells automatically indicate which value(s) have potential to increase, and which elements of Porter's strategic VBHC agenda may/should be applied.^1^

**TABLE 2:** Current practices for persons with liver GSD and FAODs and their (potential) impact at the four value‐pillars

Practice	Values			
	Personal	Technical	Allocative	Societal
IGSDPSP				+
ENGLUPRO GSDIa study (NCT04311307)	+			
FiTtINg MCADD study (NCT03761693)	+		+	
GSD communication platform	+		+	
Continuous glucose monitoring	+		+	
www.emergencyprotocol.nl	+		+	
Project ECHO	+	+	+	+
UMCG bachelor's medicine curriculum				+

*Note*: The table indicates per practice (+) which values have been impacted most directly. The open cells indicate which value may be increased by interventions based on the six elements of the strategic VBHC agenda: (1) organize into integrated practice units (IPUs); (2) measure outcomes for every patient, to improve personal value; (3) move to bundle payments for care cycles; (4) integrate care delivery across separate facilities; (5) expand excellent services across geography, and (6) build an enabling information technology platform.

Abbreviations: FAODs, fatty acid oxidation disorders; GSD, glycogen storage disease; IGSDPSP, International liver GSD Priority Setting Partnership; MCADD, medium‐chain acyl‐CoA dehydrogenase deficiency.

## 2 VALUE‐BASED RESEARCH PRACTICES

**FIGURE 1:**
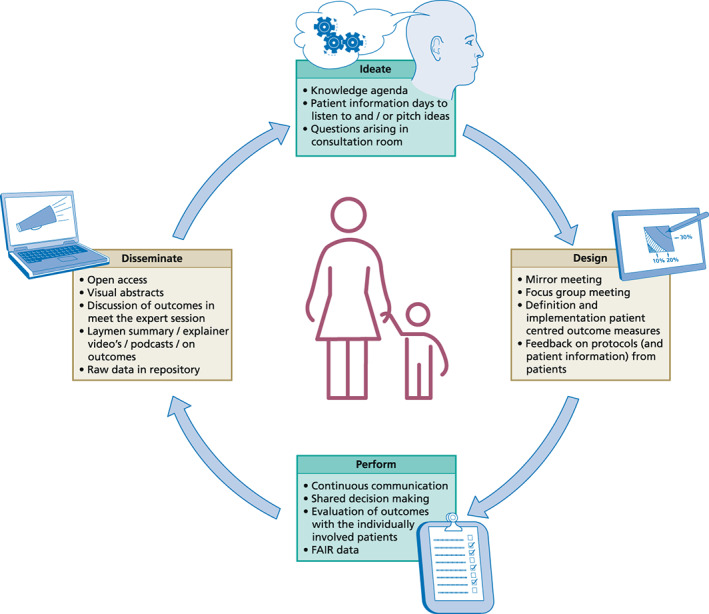
Patient involvement in all stages of research

Medical research should help healthcare providers (HCPs) and families make important decisions in the doctor's office. However, most medical research appears to be unsuitable for this, as concluded by the authors of a series of articles in The Lancet entitled “Increasing value, reducing waste.”^8^ In current biomedical research, there is waste and inefficiency in the way research is chosen, designed, performed, analyzed, regulated, managed, reported, and disseminated. Interestingly, in this series of articles the role of patients and families is hardly discussed. Adopting VBHC practices in biomedical research, may improve research in all the above‐mentioned phases of the process (Figure 1). This is illustrated by the following examples.

The International liver GSD Priority Setting Partnership (IGSDPSP) involved patient representatives worldwide in 2016–2019 to prioritize research questions for liver GSD patients that can be translated to healthcare.^9^ The IGSDPSP aimed to identify the top 10 research priorities of patients with liver GSD, their families and healthcare professionals. To reach this goal, the four‐step process from The James Lind Alliance was followed^10^: (1) gathering uncertainties, (2) formulating summary questions, (3) interim priority setting, and (4) final priority setting. This priority setting partnership involved patients, carers, and healthcare professionals from 58 countries. Out of a total of 1388 original questions from 763 responders, patients, carers, and healthcare professionals jointly agreed on a top 11 research priorities of which at least nine and five would be generalizable (after slight modifications) to the entire group of IMDs and RDs, respectively (Table 3). During the final priority setting, participants who initiated research, and thus already had the possibility to influence the research agenda, were excluded from participation. The priorities did not match those deemed by the professionals alone. Patients and carers emphasized the importance of natural progression of disease and complications. Likely, many of the original questions may have value to improve future RD research and healthcare organization.

**TABLE 3:** Research priorities for liver glycogen storage disease (GSD) patients, and the potential generalizability to other inborn metabolic diseases (IMDs) and rare diseases (RDs)

	IGSDPSP priorities	IMDs	RDs
1.	What are the best options (e.g., gene therapy or enzyme replacement therapy) for achieving sufficient amount of working enzyme in patients with liver GSD?	✓	✓
2.	Can consensus guidelines (for management) be achieved for patients with liver GSD?	✓	✓
3.	How should optimal metabolic control both clinically and biochemically (like lactate, ketones, and/or lipids) be achieved in liver GSD?	✓	
4.	How should sickness and emergency situations be managed for patients with liver GSD?	✓	✓
5.	What is the best way to start dietary treatment, finding the optimal doses, and to administer the diet for patients with liver GSD?	✓	
6.	How can existing cornstarch preparations be modified or alternative treatments be implemented that are easier to administer and/or keep blood sugar levels more stable for patients with liver GSD?	✓	
7.	What is the role for new methods for monitoring metabolic control (like noninvasive continuous glucose and lactate measurements, new biomarkers) for patients with liver GSD?	✓	✓
8.	How to manage diet regimen in relation to “before, during and after” physical exercise (sport, playing) for patients with liver GSD?	✓	✓
9.	What are the long‐term complications (liver, renal, gut) of a diet rich in uncooked cornstarch and/or high protein and should the diet be adjusted to prevent complications in liver GSD?	✓	
10.	What are the risks and benefits of different options for overnight treatment for patients with liver GSD and how can we maximize safety?		
11.	How to prevent and/or treat muscle problems in patients with liver GSD?	✓	

GSD type Ia (GSDIa; OMIM #232200) is an IMD of carbohydrate metabolism causing severe fasting intolerance.^11^ The IGSDPS emphasized the importance of improving minimally and non‐invasive monitoring modalities of metabolic control.^9^ Besides, the idea behind the ENdogenous GLUcose PROduction in patients with GSDIa (ENGLUPRO GSDIa) study (NCT04311307) grew while necessitating longitudinal and minimally invasive monitoring of outcomes,^12^ especially given the recent experimental, novel therapies. The objective of the ENGLUPRO GSDIa study is to test the feasibility of endogenous glucose production quantification in adult GSDIa subjects by stable isotopes after a single oral [6,6‐^2^H_2_]glucose dose. Patients provided feedback both to early drafts of the study protocol and the patient information leaflets before submission to the Institutional Review Board. Financial support for this study was in part granted by Associazione Italiana Glicogenosi, the Italian GSD patient organization.

Medium‐chain acyl‐CoA dehydrogenase deficiency (MCADD; #OMIM 201450) is the most common FAOD. The safe and unsafe duration of fasting in children MCADD has been retrospectively studied^13^ before the disorder has been implemented in the Dutch population newborn bloodspot screening program since 2007.^14^ In the following years, we have experienced that the recommendations for safe fasting and the subsequent night feedings and late evening feedings have important impact for the affected families. The recommendations were questioned by a subset of the parents, who sometimes accidentally found that their child could fast longer than recommended under normal circumstances. In the subsequent years, the idea behind the Fasting Tolerance in MCADD‐infants (FiTtINg MCADD) study (NCT03761693) came to attention at the doctor's office. In brief, in this investigator‐initiated pilot study with parental commitment, 2 and 6 months old MCADD infants undergo a supervised controlled clinical fasting challenge to determine their fasting tolerance. Parent's opinions have been taken into account at various developmental stages of this research project. To establish relevance of this research topic and parental role, the idea has been discussed at the Dutch patient information meeting on MCADD, hosted by the Dutch Patient Association for Inherited Metabolic Diseases (VKS). Patients' parents were included in the development of the study protocol design from the start. Before drafting the research protocol, the investigators attended a mirror meeting from a focus group, organized by the patient association. In this mirror meeting, patients' parents have discussed aspects they deemed relevant for the proposed study. This input was used in the design of the study and the researchers shared the draft study protocols and patient information forms for feedback. During study visits of this currently ongoing study, efforts are made to actively involve parents of patients in clinical decision making. Financial support for this study was in part granted by Stichting Stofwisselkracht, a fundraising organization founded by IMD parents and run entirely by volunteers.

## 3 VBHC PRACTICES

Since the 1990s, representatives of different national GSD support groups have asked expert HCPs for common sets of treatment and follow‐up procedures.^15^ Guidelines have subsequently been published: the European guidelines for GSDs Ia^16^ and GSD Ib^17^ in 2002, and the American College of Medical Genetics and Genomics guidelines for GSD III^18^ in 2010, GSD I^19^ in 2014, and GSD VI and IX^20^ in 2019, respectively. The latter guidelines were produced with research funds awarded by the American GSD patient organization (Association for GSDs). To date GSD patients themselves have not been involved in the developmental and implementation stages of these guidelines. The biomedical outcomes and thresholds that are described in these guidelines are defined by HCPs and include clinical parameters (e.g., symptoms and signs, weight, height, and liver size) and circulating biomarkers in blood, such as glucose, ketones, lactate, triglycerides, total cholesterol, and uric acid. These traditional outcomes only capture a snapshot of the patients' metabolic status when visiting the HCP but may differ from what could be observed at home.

With input from GSD patients, the GSD communication platform has been developed for individual liver GSD patients to support home monitoring.^21^ This telemedicine platform integrates biochemical, physiological, and dietary parameters and can provide patient with more detailed information on their disease, resulting in better insights and more autonomy. The platform also included continuous glucose monitoring (CGM) data. The CGM technology allows real‐time assessment of glucose management in GSD patients at home. Previous studies on the application of CGM in GSD patients have shown that CGM can help in individual patients' glucose management.^22^ Kaiser and co‐workers found that the quality of glycemic control assessed by CGM correlated with long‐term complications such as hepatocellular adenoma formation and development of microalbuminuria.^23^ The study by Peeks and co‐workers proposed more in depth CGM data analysis, and summarized recommended indications for CGM monitoring and proposed CGM outcome parameters for assessment of glucose management in persons with GSD.^24^ Subsequently, we prospectively studied CGM outcomes in adults with GSDIa, which may be useful for the definition of glycemic targets both in healthcare and clinical trials.^25^ Besides CGM, the topics of psychosocial problems and eating disorders in GSD patients^26^ and the impact of GSDI on daily, adult life^27^ have increasingly gained attention.

A common feature of GSD and FAOD is fasting intolerance. If this is not sufficiently treated or prevented, it can lead to acute life‐threatening complications, as hypoglycemia, convulsions, coma, or death. Emergency letters have been of great importance, to ensure patients' safety, and to facilitate knowledge dissemination. Rossi and co‐workers retrospectively investigated the use of emergency letters based on a generic emergency protocol in patients with liver GSD and FAOD.^28^ The website www.emergencyprotocol.net was generated in the context of the CONNECT MetabERN eHealth project involving 54 participants (HCPs and patients' representatives from 15 countries). Emergency letter availability led to appropriate initial treatment in any hospital without delay, after which metabolic expertise is sought. In addition, patients and parents can generate the emergency letters themselves therefore constituting a mean to promote patients' empowerment. As of July 29, 2022, 1912 emergency letters have been generated online in 11 languages (Dutch, *n* = 561; English, *n* = 507; French, *n* = 84; German, *n* = 181; Greek, *n* = 5; Italian, *n* = 133; Polish, *n* = 26; Portuguese, *n* = 195; Spanish, *n* = 159; Turkish, *n* = 57).

## 4 VALUE‐BASED EDUCATION AND TRAINING PRACTICES

In RDs access to highly specialized expertise can be difficult, thus leading to healthcare discrepancies and poor health outcomes. Project ECHO (https://hsc.unm.edu/echo/) is a movement whose mission is to democratize medical knowledge and amplify the capacity to provide best practice for underserved people all over the world. The ECHO model is based on four principles: (a) amplification, to use technology to leverage scarce resources; (b) best practices, to reduce disparity; (c) case‐based learning, to master complexity, and (d) data, to monitor outcomes with a web‐based database. The ECHO model provides and increases specialized medical knowledge for clinicians in primary care centers. Thereby, project ECHO acts as a forum for specialists to mentor colleagues, extend professional networks, and implement best practices and holistic care, ultimately improving patient and healthcare outcome.

A large minority of the ECHO Programs and ECHO Hubs are active in the EU. Interestingly, none of these European ECHO hubs/programs is regarding RDs. Important lessons can be learnt from ECHO programs in the RD domain, such as the electronic Genetic Nutrition Academy (since 2017),^29^ the Learn Intestinal Failure Tele‐ECHO (since 2019),^30^ and the TeleECHO program on rare bone diseases (since 2019).^31^ With the appropriate funding in place, TeleECHO programs might help to improve future healthcare for RD patients and their HCPs.

In the bachelor's curriculum in medicine at the University of Groningen, education on IMDs has incorporated storytelling in collaboration with patients. This patient‐centered approach allows students from the start of their education to experience the authentic story of the patient and his family. In addition to patient lectures, a digital platform is created with four patient‐centered e‐learning cases. The Journal of Inherited Metabolic Diseases “View from Inside” articles are provided as additional study materials.^32^, ^33^ In this way, student‐patient interaction is encouraged.

## 5 SUMMARY AND FUTURE PERSPECTIVES

VBHC is challenging to apply to low volume, high complex healthcare situations. The current practices for persons with GSD or FAOD exemplify how value can been increased at multiple academic domains: science, expert healthcare, and education and training. How can we move forward to a high‐value healthcare delivery system for persons with IMDs?

Porter and Lee identified six elements of the strategic VBHC agenda, that are interdependent and mutually reinforcing^1^:

1. organize into integrated practice units (IPUs);
2. measure outcomes for every patient, to improve personal value;
3. move to bundle payments for care cycles;
4. integrate care delivery across separate facilities;
5. expand excellent services across geography, and
6. build an enabling information technology platform.

**FIGURE 2:**
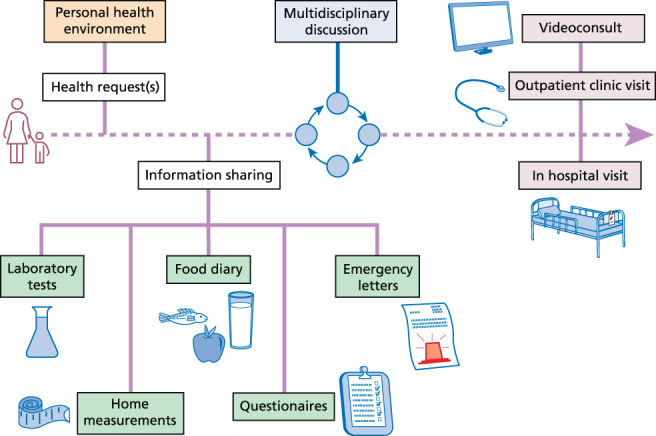
Health request driven and person‐centered organizational care support pathway. Data from the electronic health records (in orange, including the prescribed diet), should ideally be fully interoperable with home measurements (in green, including laboratory values, continuous glucose monitoring, and food diaries, i.e., the actual diet). Based on these incoming data, it can be discussed in a multidisciplinary setting (in blue), whether the medical expertise can be transferred by telemedicine, or if hospital visit is required (in purple).

Progress will be easiest and fastest if these elements are advanced together, improving individuals' healthcare in a health request driven manner, aiming to deliver the right care, at the right time, for the right person (Figure 2).

The IGSDPSP has mostly generated “societal value,” as the partnership reached out to the international community to prioritize research topics for the field of liver GSD. Thereby, the IGSDPSP has provided guidance for those influencing the research agenda and future decisions about resource allocation. At the first place in the top 11 priorities for research, the IGSDPSP identified the question “What are the best options (e.g., gene therapy or enzyme replacement therapy) for achieving sufficient amount of working enzyme in patients with liver GSD?”. Interestingly, this seems very similar to the top one priority in the recent priority setting partnership for people with mitochondrial diseases (e.g., “Could an understanding of the cellular and molecular processes in mitochondrial disease lead to new therapies?”).^34^ Therapy development seems to be a common priority for IMDs, perhaps even for RDs in general. The challenge is how to involve all stakeholders (including consumers) to feel responsible for the stewardship of resources to optimize “technical value” and “allocative value.”

At the second place in the top 11 priorities for research in liver GSD, the IGSDPSP prioritized the question “Can consensus guidelines (for management) be achieved for patients with liver GSD?” Previous GSD guidelines and care pathway publications have been largely based on expert opinions. These guidelines and care pathways do not systematically cover CGM parameters, patient‐reported outcome measures (PROMs) and patient‐reported experience measures (PREMs), and have lacked participation by families and patients. Measurement of outcomes for individual patient directly improves “personal value” and requires to define and measure standardized and person‐centered outcomes, including PROMs/PREMs, in order to improve the care for individual patients and to enable patients to report directly on their disease.

Patients and families collaborated in the telehealth practices (GSD communication platform, CGM, www.emergencyprotocol.net), which most directly generated “personal value” given the level of self‐management and self‐monitoring of persons with GSD and FAOD. The open character of www.emergencyprotocol.net adds “technical value” and “allocative value.” These telehealth practices' abilities to increase “societal value” has been challenged by technical, societal, medical, and legal developments, as discussed previously.^21^ Important lessons can be learnt from diabetes mellitus, the most prevalent disorder in which glucose homeostasis is perturbed. Recent experiences with healthcare organization for people with diabetes mellitus exemplify, how telehealth may expand excellent services across geography.^35^ Building an enabling information technology platform and bundle payments for care cycles have been fundamental in the case of the Dutch treatment center Diabeter.^36^ In addition, recently, Nano and co‐workers reported on a standard set of outcome measures for diabetes mellitus that included PROMs and PREMs, in collaboration with the International Consortium for Health Outcomes Measurement.^37^ Some elements of this standard set of outcomes can be applied to persons with GSD and FAOD, such as the number of acute hospital admissions or hypoglycemias, or the CGM parameters. Standard sets of person‐centered outcome parameters are driven by medical needs and should include (disease specific) PROMs and PREMs. Currently, standard sets are in preparation for few IMDs, such as mucopolysaccharidoses,^38^ phenylketonuria and MCADD.^39^ International consensus on outcomes is a prerequisite for (a) evaluation of individual healthcare in PDCA (Plan‐Do‐Check‐Act) cycles, (b) international data collection, (c) sustainable rare disease registries, and (d) innovative orphan drug development. Since 2011, the COMET (Core Outcome Measures in Effectiveness Trials) initiative (www.comet-initiative.org) has evolved to promote the performance of literature reviews and multi‐stakeholder consensus approaches (including patient involvement) in the selection of outcomes for clinical trials.^40^ Unfortunately, only a handful of IMDs is currently registered in the COMET database and the use of PROMs as endpoints in clinical trials for RDs is severely lacking.^41^

In the VBHC approach, healthcare delivery is organized in so‐called IPU, that are defined as “a unit organized around the patient that provides the full cycle of care for a medical condition, including patient education, engagement, and follow‐up. It also encompasses inpatient, outpatient and rehabilitative care as well as supporting services.” At a state or national level, multiple HCPs, institutions, and geographic areas can be involved in the full cycle of care of an individual patient. The healthcare institutions are typically paid per activity rather than being rewarded “for doing nothing,” cost savings or healthcare innovations. Reimbursements are not yet in place for innovative healthcare services that are important for families with RDs and IMDs, such as virtual counseling, providing structured expert HCP opinions or panel discussions, or innovative drug repurposing. In many areas, expert HCPs depend on institutional investments for their practices, grants, contracts with private parties, or philanthropy. Since 2017, European Reference Networks have been established for RDs, among which the European Reference Network for Hereditary Metabolic Disorders (MetabERN). A recent survey by the MetabERN revealed a widespread lack of social, psychological, and economic support for IMD patients in Europe.^42^ In theory, digitalization may allow expanding healthcare expertise in a cross‐border manner and European Reference Networks, to function as so‐called network IPUs. It is yet a question whether these European Reference Networks may, can and will act as sustainable, virtual IPUs, paving the way toward bundle payments for expert care cycles. Within any virtual IPU, project ECHO has the potential to increase value at all value‐pillars, including “personal value” to both patients and HCPs, by propagating case‐based learning.

Data repurposing is warranted for RDs, which is also demonstrated for electronic health record data for the diagnosis of RDs.^43^ Ideally, real‐world data and routinely collected healthcare data should be processed according to the FAIR guiding Principles (Findability, Accessibility, Interoperability, and Reusability), as exemplified by the Swiss approach.^44^ Governance on individuals' big health data and their interoperability should become top priorities. Sustainable coalitions are necessary between all relevant stakeholders, such as HCPs, institutions, payers, industrial parties, insurance companies, healthcare planning and health policy, and governments, putting patients and their families at the center.

Important developments have occurred in the field of IMD medicine in the last decades. There have been profound technological advancements in diagnosis and treatment. Thanks to the expansion of the population newborn screening programs and the introduction of next generation sequencing techniques, the diagnostic odyssey for many families with IMDs (and other RDs) has been importantly reduced. The increased connectivity via social media has positive effects on disease awareness and the spread of knowledge, leading to a change in the relationships between HCPs and patients, and hence, increasing patients' and family's empowerment and engagement. Emerging innovative treatment strategies are becoming available for an increasing number of RDs, including organ transplantations and genetic therapies.^45^ These developments emphasize both the need and the potential to apply VBHC principles to IMDs.

To conclude, for low volume, high complex healthcare situations there are specific difficulties that can be overcome by adopting VBHC principles. The current practices for people with GSD and FAOD have taken place in all academical domains (research, healthcare, education, and training). VBHC principles and strategic agenda points can support the organization of personalized medicine (personal value) and subsequently generate value at four value‐pillars.

## AUTHOR CONTRIBUTIONS

Presented as “Consumer consultation to provide focused family centered‐care; based on some value‐based healthcare principles” at the 14th International Congress of Inborn Errors of Metabolism 2021 Transforming Rare Disorders” in Sydney, Australia, November 23, 2021 by Terry G. J. Derks. All authors contributed to data collection and revised the manuscript for important intellectual content. All authors approved the final manuscript as submitted and agreed to be accountable for all aspects of the work. All authors confirm the absence of previous similar or simultaneous publications.

## FUNDING INFORMATION

The MD‐PhD scholarships of Annieke Venema (MD‐PhD 21‐65), Fabian Peeks (MD‐PhD 16‐24), and Emmalie A. Jager (MD‐PhD 18‐55), are funded by the Junior Scientific Masterclass from the University of Groningen, University Medical Center Groningen. Alessandro Rossi was funded by Associazione Italiana Glicogenosi (Grant/Award Number: 01/2020).

## CONFLICT OF INTEREST

All authors declare that they have no conflict of interest related to this manuscript.

## ETHICS STATEMENT

All procedures followed were in accordance with the ethical standards of the responsible committee on human experimentation (institutional and national) and with the Helsinki Declaration of 1975, as revised in 2000.

## Data Availability

My manuscript has no associated data.

## References

[1] Porter ME . A strategy for health care reform—toward a value‐based system. N Engl J Med. 2009;361:109‐112. 19494209 10.1056/NEJMp0904131

[2] Expert Panel on effective ways of investing in Health (EXPH) Defining value in “value‐based healthcare”. 2019.

[3] Augustine EF , Dorsey ER , Saltonstall PL . The care continuum: an evolving model for care and research in rare diseases. Pediatrics. 2017;140(3):e20170108. 28818836 10.1542/peds.2017-0108

[4] Fantini B , Vaccaro CM . Value based healthcare for rare diseases: efficiency, efficacy, equity. Ann Ist Super Sanita. 2019;55:251‐257. 31553319 10.4415/ANN_19_03_10

[5] Kole A , Hedley V . Recommendations from the Rare 2030 Foresight Study: the future of rare diseases starts today [Internet]. 2021. https://www.rare2030.eu/recommendations/

[6] Weinstein DA , Steuerwald U , de Souza CFM , Derks TGJ . Inborn errors of metabolism with hypoglycemia: glycogen storage diseases and inherited disorders of gluconeogenesis. Pediatr Clin North Am. 2018;65(2):247‐265. 29502912 10.1016/j.pcl.2017.11.005

[7] Houten SM , Violante S , Ventura FV , Wanders RJA . The biochemistry and physiology of mitochondrial fatty acid β‐oxidation and its genetic disorders. Annu Rev Physiol. 2016;78:23‐44. 26474213 10.1146/annurev-physiol-021115-105045

[8] Macleod MR , Michie S , Roberts I , et al. Biomedical research: increasing value, reducing waste. Lancet. 2014;383(9912):101‐104. 24411643 10.1016/S0140-6736(13)62329-6

[9] Peeks F , Boonstra WF , de Baere L , et al. Research priorities for liver glycogen storage disease: an international priority setting partnership with the James Lind Alliance. J Inherit Metab Dis. 2020;43(2):279‐289. 31587328 10.1002/jimd.12178 PMC7079148

[10] James Lind Alliance [Internet]. https://www.jla.nihr.ac.uk/jla-guidebook/

[11] Moses SW , Shin YS , Smit GPA , Ullrich K . Glycogen storage disease type I: preface/introduction. Eur J Pediatr. 1993;152(Suppl 1):1‐28.8444197

[12] Derks TGJ , Oosterveer MH , de Souza CF . Next‐generation glycogen storage diseases. J Inherit Metab Dis. 2018;41(6):911‐912. 30471000 10.1007/s10545-018-00250-0 PMC6326968

[13] Derks TGJ , van Spronsen FJ , Rake JP , van der Hilst CS , Span MM , Smit GPA . Safe and unsafe duration of fasting for children with MCAD deficiency. Eur J Pediatr. 2007;166(1):5‐11. 16788829 10.1007/s00431-006-0186-0

[14] Crespo Marcos D , López‐Menchero Oliva JC , Carreño Beltrán A , Rodríguez Fernández R , Rodríguez SA . Medium‐chain acyl‐coenzyme a dehydrogenase deficiency. An Pediatr (Engl Ed). 2008;68(3):302‐304. 10.1157/13116715 18358146

[15] Phillips A . More questions: 10 years later. Eur J Pediatr. 2002;161:S102‐S105. 12373582 10.1007/BF02680005

[16] Rake JP , Labrune P , Ullrich K , Smit P , Visser G , Leonard J . Guidelines for management of glycogen storage disease type I ‐ European Study on Glycogen Storage Disease Type I (ESGSD I). Eur J Pediatr. 2002;161:S112‐S119. 12373584 10.1007/s00431-002-1016-7

[17] Visser G , Rake J , Labrune P , et al. Consensus guidelines for management of glycogen storage disease type 1b ‐ European Study on Glycogen Storage Disease Type 1. Eur J Pediatr. 2002;161:S120‐S123. 12373585 10.1007/s00431-002-1017-6

[18] Kishnani PS , Austin SL , Arn P , et al. Glycogen storage disease type III diagnosis and management guidelines. Genet Med. 2010;12(7):446‐463. 20631546 10.1097/GIM.0b013e3181e655b6

[19] Kishnani PS , Austin SL , Abdenur JE , et al. Diagnosis and management of glycogen storage disease type I: a practice guideline of the American College of Medical Genetics and Genomics. Genet Med. 2014;16(11):1‐29. 10.1038/gim.2014.128 25356975

[20] Kishnani PS , Goldstein J , Austin SL , et al. Diagnosis and management of glycogen storage diseases type VI and IX: a clinical practice resource of the American College of Medical Genetics and Genomics (ACMG). Genet Med. 2019;21(4):772‐789. 30659246 10.1038/s41436-018-0364-2

[21] Hoogeveen IJ , Peeks F , de Boer F , et al. A preliminary study of telemedicine for patients with hepatic glycogen storage disease and their healthcare providers: from bedside to home site monitoring. J Inherit Metab Dis. 2018;41(6):929‐936. 29600495 10.1007/s10545-018-0167-2 PMC6326981

[22] Kasapkara CS , Cinasal Demir G , Hasanoǧlu A , Tümer L . Continuous glucose monitoring in children with glycogen storage disease type I. Eur J Clin Nutr. 2014;68(1):101‐105. 24149443 10.1038/ejcn.2013.186

[23] Kaiser N , Gautschi M , Bosanska L , et al. Glycemic control and complications in glycogen storage disease type I: results from the Swiss registry. Mol Genet Metab. 2019;126(4):355‐361. 30846352 10.1016/j.ymgme.2019.02.008

[24] Peeks F , Hoogeveen IJ , Feldbrugge RL , et al. A retrospective in‐depth analysis of continuous glucose monitoring datasets for patients with hepatic glycogen storage disease: recommended outcome parameters for glucose management. J Inherit Metab Dis. 2021;44(5):1136‐1150. 33834518 10.1002/jimd.12383 PMC8519135

[25] Rossi A , Venema A , Haarsma P , et al. A prospective study on continuous glucose monitoring in glycogen storage disease type Ia: toward glycemic. Targets. 2022;107:1‐12. 10.1210/clinem/dgac411 PMC9387687 35786777

[26] Venema A , Peeks F , Bruijn‐van der Veen M , et al. A retrospective study of eating and psychosocial problems in patients with hepatic glycogen storage diseases and idiopathic ketotic hypoglycemia: towards a standard set of patient‐reported outcome measures. JIMD Rep. 2021;63:29‐40. 35028269 10.1002/jmd2.12253 PMC8743343

[27] Garbade SF , Ederer V , Burgard P , et al. Impact of glycogen storage disease type I on adult daily life: a survey. Orphanet J Rare Dis. 2021;16(1):1‐10. 10.1186/s13023-021-02006-w 34479584 PMC8414849

[28] Rossi A , Hoogeveen IJ , Lubout CMA , et al. A generic emergency protocol for patients with inborn errors of metabolism causing fasting intolerance: a retrospective, single‐center study and the generation of www.emergencyprotocol.net. J Inherit Metab Dis. 2021;44(5):1124‐1135. 33844307 10.1002/jimd.12386 PMC8518720

[29] electronic Genetic Nutrition Academy [Internet]. 2017. Accessed September 5, 2022. https://egeneticnutritionacademy.org/

[30] Winkler MF , Tappenden KA , Spangenburg M , Iyer K . Learn intestinal failure tele‐ECHO project: an innovative online telementoring and case‐based learning clinic. Nutr Clin Pract. 2021;36(4):785‐792. 34159643 10.1002/ncp.10743

[31] Tosi LL , Rajah EN , Stewart MH , Gillies AP , Hart TS , Lewiecki EM . The rare bone disease TeleECHO program: leveraging telehealth to improve rare bone disease care. Curr Osteoporos Rep. 2020;18(4):344‐349. 32514667 10.1007/s11914-020-00595-2 PMC7419447

[32] Halai J . View from inside. J Inherit Metab Dis. 2018;41(6):901‐903. 29967950 10.1007/s10545-018-0214-z

[33] View from inside: Nina, glycogen storage disease warrior. J Inherit Metab Dis. 2020;43(4):653‐656. 32379349 10.1002/jimd.12246

[34] Thomas RH , Hunter A , Butterworth L , et al. Research priorities for mitochondrial disorders: current landscape and patient and professional views. J Inherit Metab Dis. 2022;45:796‐803. 35543492 10.1002/jimd.12521 PMC9429991

[35] Crossen S , Raymond J , Neinstein A . Top 10 tips for successfully implementing a diabetes telehealth program. Diabetes Technol Ther. 2020;22(12):920‐928. 32191141 10.1089/dia.2020.0042 PMC7757601

[36] Diabeter [Internet]. Accessed August 2, 2022. https://diabeter.nl/en

[37] Nano J , Carinci F , Okunade O , et al. A standard set of person‐centred outcomes for diabetes mellitus: results of an international and unified approach. Diabet Med. 2020;37(12):2009‐2018. 32124488 10.1111/dme.14286

[38] Howie AH , Tingley K , Inbar‐Feigenberg M , et al. Establishing a core outcome set for mucopolysaccharidoses (MPS) in children: study protocol for a rapid literature review, candidate outcomes survey, and Delphi surveys. Trials. 2021;22(1):1‐11. 34789302 10.1186/s13063-021-05791-8 PMC8600749

[39] Potter BK , Hutton B , Clifford TJ , et al. Establishing core outcome sets for phenylketonuria (PKU) and medium‐chain Acyl‐CoA dehydrogenase (MCAD) deficiency in children: study protocol for systematic reviews and Delphi surveys. Trials. 2017;18(1):1‐10. 29258568 10.1186/s13063-017-2327-3 PMC5735866

[40] Williamson PR , Altman DG , Bagley H , et al. The COMET handbook: version 1.0. Trials. 2017;18(Suppl 3):1‐50. 28681707 10.1186/s13063-017-1978-4 PMC5499094

[41] Rubinstein YR , Robinson PN , Gahl WA , et al. The case for open science: rare diseases. JAMIA Open. 2020;3(3):472‐486. 33426479 10.1093/jamiaopen/ooaa030 PMC7660964

[42] Sestini S , Paneghetti L , Lampe C , et al. Social and medical needs of rare metabolic patients: results from a MetabERN survey. Orphanet J Rare Dis. 2021;16(1):1‐13. 34344397 10.1186/s13023-021-01948-5 PMC8329639

[43] Garcelon N , Burgun A , Salomon R , Neuraz A . Electronic health records for the diagnosis of rare diseases. Kidney Int. 2020;97(4):676‐686. 32111372 10.1016/j.kint.2019.11.037

[44] Rakic M , Jaboyedoff M , Bachmann S , et al. Clinical data for paediatric research: the Swiss approach: proceedings of the National Symposium in Bern, Switzerland, Dec 5‐6, 2019. BMC Proc. 2021;15(Suppl 13):1‐15. doi:10.1186/s12919-021-00226-3 34538238 PMC8450032

[45] Bick D , Bick SL , Dimmock DP , Fowler TA , Caulfield MJ , Scott RH . An online compendium of treatable genetic disorders. Am J Med Genet C Semin Med Genet. 2021;187(1):48‐54. 33350578 10.1002/ajmg.c.31874 PMC7986124

